# Frequency difference mapping applied to the corpus callosum at 7T

**DOI:** 10.1002/mrm.27626

**Published:** 2018-12-23

**Authors:** Benjamin C. Tendler, Richard Bowtell

**Affiliations:** ^1^ Sir Peter Mansfield Imaging Centre, School of Physics and Astronomy University of Nottingham United Kingdom; ^2^ Wellcome Centre for Integrative Neuroimaging, FMRIB, Nuffield Department of Clinical Neurosciences University of Oxford Oxford United Kingdom

**Keywords:** corpus callosum, frequency difference mapping, microstructure, phase processing, three‐pool model, white matter

## Abstract

**Purpose:**

Frequency difference mapping (FDM) is a phase processing technique which characterizes the nonlinear temporal evolution of the phase of gradient echo (GE) signals. Here, a novel FDM‐processing algorithm is introduced, which is shown to reveal information about white matter microstructure. Unlike some other phase‐processing techniques, the FDM algorithm presented here does not require the use of phase unwrapping or sophisticated image processing. It uses a series of scaled complex divisions to unwrap phase and remove background fields.

**Methods:**

Ten healthy subjects underwent a series of single‐slice, sagittal multi‐echo GE scans at 7T with the slice positioned at the midline. Phase data were processed with the novel FDM algorithm, and the temporal evolution of the magnitude signal and frequency difference was examined in 5 regions of the corpus callosum (CC; genu, anterior body, middle body, posterior body, and splenium).

**Results:**

Consistent frequency difference contrast relative to surrounding tissue was observed in all subjects in the CC and in other white matter regions where the nerve fibers run perpendicular to $B_{0}$ *,* such as the superior cerebellar peduncle. Examination of the frequency difference curves shows distinct variations over the CC, with the genu and splenium displaying larger frequency differences than the other regions (in addition to a faster decay of signal magnitude).

**Conclusion:**

The novel FDM algorithm presented here yields images sensitive to tissue microstructure and microstructural differences over the CC in a simple manner, without the requirement for phase unwrapping or sophisticated image processing.

## 1 INTRODUCTION

Access to higher field strengths and improved processing techniques has opened up new opportunities for exploiting the contrast provided by the phase of gradient echo (GE) images.^1^, ^2^ Phase images are already being widely used in clinical studies based upon susceptibility weighted imaging^3^—a technique which combines magnitude data and high‐pass filtered phase images to improve image contrast, particularly around veins. Quantitative susceptibility mapping (QSM), in which maps of susceptibility variation are derived from phase images, is also now being utilized in clinical investigations,^4^, ^5^ and QSM has been extended to mapping of the anisotropy of magnetic susceptibility in susceptibility tensor imaging (STI).^6^ Calculation of maps showing the variation of isotropic or anisotropic magnetic susceptibility generally involves phase unwrapping and background field removal, followed by deconvolution using the appropriate dipole field kernel.^5^

QSM and STI both rely on the assumption that the phase measured in a voxel is linearly related to the TE and the local field perturbation, and that after background field removal this field perturbation can be described as the sum of the dipole field contributions from all the voxels in the volume of interest, with each contribution depending upon the average magnetic susceptibility of the material within the voxel. Recent work has, however, shown that phase evolution in white matter is nonlinear with TE^7^, ^8^, ^9^, ^10^ and that maps of the phase variation in the brain cannot be completely described by simply summing the dipole fields produced by the average magnetization in each voxel.^11^, ^12^ This behavior occurs in the presence of subvoxel microstructure, which gives rise to a heterogeneous and anisotropic distribution of magnetic susceptibility that is accompanied by a variation of the nuclear magnetic resonance (NMR) signal strength from different compartments of susceptibility distribution. Under these circumstances, the signal from a voxel is a weighted sum of terms evolving at different frequencies,^7^, ^8^, ^10^, ^13^ and the signal phase does not necessarily reflect the average field offset in the voxel.

In white matter, the relevant microstructure arises from the effect of the myelin sheaths of coherently oriented nerve fibers. These have an anisotropic magnetic susceptibility, described by a susceptibility tensor with a radially oriented principal axis,^7^ along with a low water content and short *T*_2_^*^ 8, 10 relative to the axonal and interstitial compartments. As a consequence, NMR signals from the myelin water between the myelin lipid bilayers and the axonal compartment both have average frequency offsets relative to the interstitial water (here denoted as the external compartment) that depend on fiber geometry, including orientation of nerve fibers with respect to the applied magnetic field, $B_{0}$,^9^, ^14^, ^15^ with a smaller relative signal from the myelin water than would be predicted from the myelin volume fraction, and a signal that decreases rapidly with TE. Sensitivity of signal evolution to the local nerve fiber architecture produces a local, microstructure‐related contribution to the phase that is not represented by the dipole field kernels used in QSM and STI, and hence gives rise to errors in the resulting susceptibility maps.^11^ More positively, this sensitivity opens up a new way of probing the microstructure of white matter. The effect of microstructure on GE signals is largest when nerve fibers are oriented perpendicular to the applied field.^7^

To assess the effects of microstructure on GE images, evolution of the signal from white matter is best described using a three‐pool model in which the external, myelin, and axonal compartments each have different signal amplitudes, decay rates, and frequency offsets.^8^ This model has been used to analyze multi‐echo GE data in a range of applications, including improved mapping of the myelin water fraction using GE imaging,^16^ evaluation of axonal density in postmortem tissue,^17^ evaluating echo‐time dependence of QSM measurements^18^, ^19^ and assessing microstructural variation in the corpus callosum (CC).^20^ To reveal the effects of tissue microstructure on the evolution of signal phase, it is necessary to remove the much larger TE‐dependent phase offsets resulting from field variation produced by nonlocal sources, as well as other non‐TE‐dependent phase variation—for example, because of radiofrequency (RF) interaction with the tissue.^21^ This can be done by fitting the temporal evolution of the signal on a pixel‐wise basis or in small regions of interest (ROIs), but calculation of the change in the rate of phase evolution with TE also offers a simple approach to mapping microstructural effects. In its simplest form, this frequency difference mapping (FDM) involves evaluating the difference in the rate of phase accumulation at short and long TEs in a multi‐echo GE data set.^22^ Sensitivity of the frequency difference measured in white matter to fiber orientation with respect to the field has previously been demonstrated in in‐vivo experiments carried out on human subjects at 7T^7^, ^8^, ^10^ and confirmed in experiments on postmortem samples from the porcine^9^ and human brain.^14^

Previous implementations of FDM have generally involved significant image processing to eliminate the effects of field variation produced by nonlocal sources and RF phase effects, including phase unwrapping, background field removal, and/or high‐pass filtering.^7^, ^8^, ^9^, ^20^, ^22^ Here, we describe a simplified approach to FDM which allows images that are sensitive to the local white matter microstructure to be rapidly generated using complex image division.^23^ This approach has been applied to imaging of the CC in 10 healthy subjects. In addition to showing FDM results, we also report the results produced by fitting the FDM and magnitude signal evolution with TE to the three‐pool model. The CC was selected for study because it is a large white matter region in which fibers are coherently oriented perpendicular to $B_{0}$, when the subject is supine inside the scanner, thus producing the largest frequency offsets in the myelin and axonal compartments.^7^, ^8^, ^13^ In addition, variation of axonal properties along the CC has previously been investigated using histology and diffusion‐weighted imaging in MRI.^24^, ^25^

## 2 THEORY

The evolution with time, t, of the complex signal recorded from a white matter voxel in a GE experiment can be written as Equation 1:

(1) $$S\left(t\right)=S_{0}e^{i\phi_{0}}e^{i\Omega t}F\left(t\right),$$

where $S_{0}$ is the signal amplitude, Ω represents the effect of the average frequency offset in the voxel, which includes the effect of fields produced by sources outside the voxel, and $\phi_{0}$ represents the time‐independent phase offset that relates to instrumental factors and RF phase effects. F(t) characterizes the effects of microstructure on the complex signal evolution. It describes the effects of interference and decay of the signals from the different compartments, producing the microstructure‐dependent, nonlinear phase evolution which is characterized in FDM. Using the three‐pool model of white matter,^8^, ^9^, ^10^ we can write Equation 2:

(2) $$F\left(t\right)=A_{a}e^{i2\pi f_{a}t-\frac{t}{T_{2a}^{*}}}+A_{m}e^{i2\pi f_{m}t-\frac{t}{T_{2m}^{*}}}+A_{e}e^{i2\pi f_{e}t-\frac{t}{T_{2e}^{*}}},$$

where subscripts a, m, and e denote the axonal, myelin, and external pools, $A_{a,m,e}$ represent the relative signal amplitudes, $T_{2a,m,e}^{*}$ are the characteristic $T_{2}^{*}$ relaxation times, and $f_{a,m,e}$ represent the frequency offsets of the different pools.

To reveal the microstructural effects manifested in the phase of F(t), we need to eliminate the much larger phase variations related to the effects of $\phi_{0}$ and Ω. This can potentially be done by voxel‐wise fitting to the phase of Equation 1,^16^ but this generally requires implementation of phase unwrapping and use of a multiparametric fit. A simpler approach is to exploit the simple linear scaling of phase effects resulting from Ω in a standard multi‐echo GE sequence in which the echoes are equally spaced in time. In such a sequence with initial TE, ${TE}_{1}$, and an interecho spacing, ΔTE, such that the n ^th^ TE is ${{TE}_{n}=TE}_{1}+\left(n-1\right)\Delta TE$, then the effect of $\phi_{0}$ can be eliminated by dividing the complex signal at each TE by $S\left({TE}_{1}\right)$, giving Equation 3:

(3) $$S^{\prime }\left(TE_{n}\right)=\frac{S\left(TE_{n}\right)}{S\left(TE_{1}\right)}=e^{i\Omega (n-1)\Delta TE}\times \frac{F\left(TE_{n}\right)}{F\left(TE_{1}\right)}.$$

The effect of Ω can then be eliminated by dividing $S^{\prime }\left(TE_{n}\right)$ by ${\left(S^{\prime }\left(TE_{2}\right)\right)}^{n-1}$ for n>1 (Equation 4):

(4) $$S^{\prime \prime }\left(TE_{n}\right)=\frac{S^{\prime }\left(TE_{n}\right)}{{\left(S^{\prime }\left(TE_{2}\right)\right)}^{n-1}}=\frac{F\left({TE}_{n}\right)}{F\left({TE}_{1}\right)}\times {\left(\frac{F\left({TE}_{1}\right)}{F\left({TE}_{2}\right)}\right)}^{n-1},$$

yielding a signal which only depends on the microstructure‐related term. To generate the frequency difference map at the n ^th^ TE, we then evaluate (Equation 5):

(5) $$FDM\left(TE_{n}\right)=\frac{arg\left[S^{\prime \prime }\left(TE_{n}\right)\right]}{2\pi \left(TE_{n}-TE_{2}\right)}.$$

Inspection of Equation 5 shows that (Equation 6):

(6) $$FDM\left(TE_{n}\right)=\frac{a\text{rg}\left[\frac{\text{F}\left({\text{TE}}_{\text{n}}\right)}{\text{F}\left({\text{TE}}_{\text{1}}\right)}\right]-arg\left[{\left(\frac{F\left({TE}_{2}\right)}{F\left({TE}_{1}\right)}\right)}^{n-1}\right]}{2\pi \left(TE_{n}-TE_{2}\right)}$$

which depends on the difference in the phase accumulation between the n ^th^ echo and the first echo, compared with the phase which would be predicted to accumulate over that time by scaling the phase difference between the first and second echoes. Dividing this phase difference by $2\pi \left(TE_{n}-TE_{2}\right)$ yields the difference in the frequency predicted from the n ^th^ echo and the second echo, that is, a frequency difference map, which is automatically 0 for n=2 (and undefined for n=1). Because of the relatively short $T_{2}^{*}$ of the myelin water, a negative frequency difference is expected in the CC and other similarly oriented fiber tracts, because the signal from the myelin compartment decreases rapidly with TE and experiences a positive frequency offset in nerve fibers that are perpendicular to $B_{0}$.^7^

**Figure 1:**
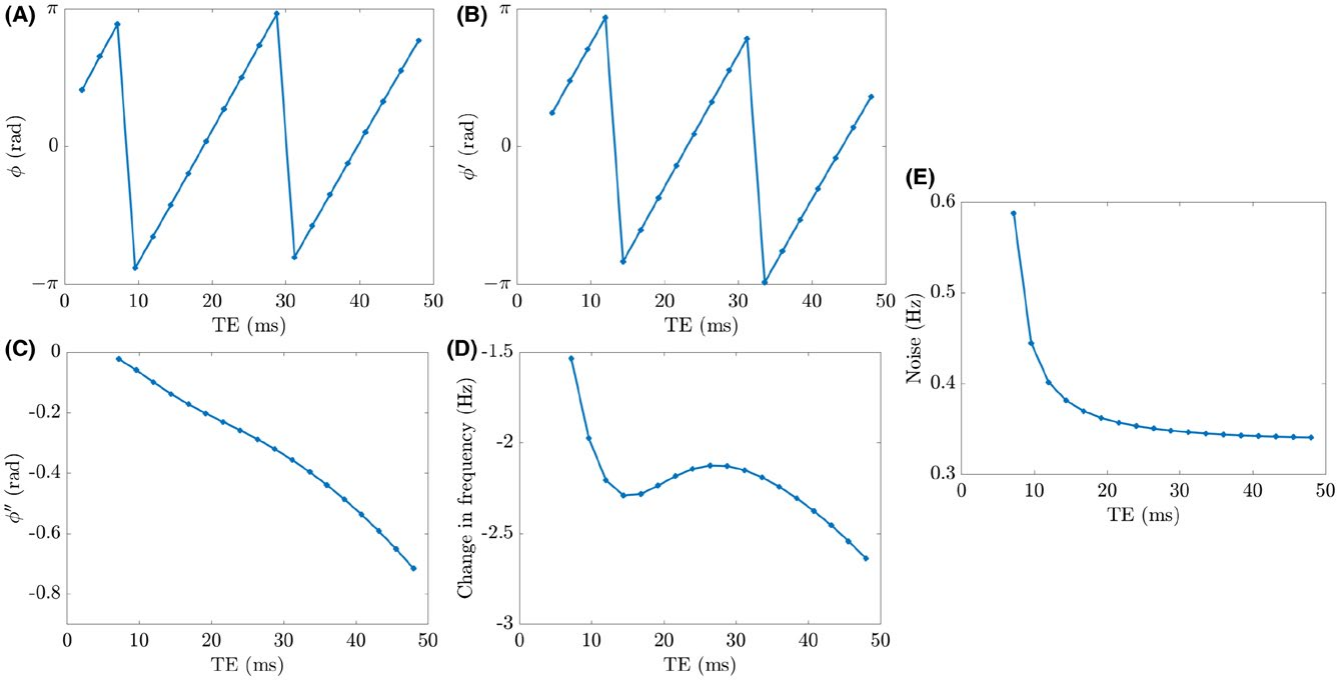
Visualization of FDM processing steps for the single‐voxel phase evolution of a triple‐exponential signal with an additional frequency offset, Ω/2π=50Hz, and a time‐independent phase offset, $\phi_{0}=0.5rad$. Parameters for the three‐pool model used in this simulation are those previously found in the splenium of the human CC in vivo at 7T^8^ and we have defined $TE_{1}=2.4ms$, ΔTE=2.4ms, and the number of echoes =20. Starting with the unprocessed wrapped phase (A), the data set is divided by the complex signal from the first echo (B and Equation 3) to remove any dependence on $\phi_{0}$. The data set is then divided by the scaled second echo signal (C and Equation 4) to remove any dependence on Ω and subsequently scaled by $2\pi ({TE}_{n}-TE_{2})$ to yield a frequency difference in Hz (D). (E) shows how the noise in the FDM signal scales with echo time (generated from Equation 7, where we have defined $T_{2}^{*}=30ms$ and $SNR_{1}=300$)

Figure 1A to D shows how the variation of phase with TE is affected by the FDM‐processing steps for the typical variation of F(t) in the CC^8^ (Ω/2π=50Hz; $\phi_{0}=0.5rad$). It is evident that the effect of the microstructure is completely obscured by the effect of Ω in the raw phase data, but shows a clear signature in the data after FDM processing.

Analysis of Equations 1 and 3 to 5 (see Supporting Information) shows that the noise in the FDM measure at the n^th^ echo time is approximately given by Equation 7:

(7) $$noise\left({TE}_{n}\right)={\left(2\pi \Delta TE\;{SNR}_{1}\right)}^{-1}\sqrt{{\left(\frac{e^{\left(n-1\right)R_{2}^{*}\Delta TE}}{n-2}\right)}^{2}+1+{\left(\frac{\left(n-1\right)e^{R_{2}^{*}\Delta TE}}{n-2}\right)}^{2}},$$

where $R_{2}^{*}$ describes the average relaxation rate of the magnitude signal and ${SNR}_{1}$characterises the signal‐to‐noise ratio (SNR) of the magnitude signal recorded at the first TE ($TE_{1}$). Figure 1E shows how the noise level in Hz evolves with TE for ΔTE=2.4ms, $R_{2}^{*}=30ms$, and $SNR_{1}=300$.

## 3 METHODS

### 3.1 Data acquisition

Using a Philips Achieva 7T MR scanner (Philips, Best, The Netherlands), 10 healthy subjects (5 males/5 females; age range, 22–25 years) underwent a series of single‐slice, sagittal multi‐echo GE scans (slice thickness =5mm, in‐plane resolution $=1\times 1{mm}^{2}$, field of view [FOV] $=224\times 224mm^{2}$, $TE_{1}=2.4ms$, ΔTE=2.4ms, number of echoes =20, TR=140ms, flip angle $=25^{\circ }$, number of averages =10, acquisition time =314seconds). We chose to acquire images of a single, relatively thick slice to provide a high SNR, and also to allow the acquisition of multiple data sets in a reasonable time, so that we could assess the errors in the frequency difference measurements. No flow compensation was applied, and all echoes were acquired under a read gradient of the same polarity, which was applied in the foot‐head direction. Scanning was approved by the local ethics committee, and all subjects gave informed consent. The sagittal slice was positioned on the midline, thus spanning a portion of the CC where the fibers are oriented perpendicular to $B_{0}$. Six scans were acquired per subject with subjects moved out and back into the scanner after the third scan to allow the repeatability between both scans and scanning sessions to be assessed. Magnitude and phase data were obtained for each subject, and phase data were processed as detailed in the Theory section to generate frequency difference maps. Both sets of 3 scans (before/after subjects were moved out and back into the scanner) were coregistered to the first of the 3 scans using FSL FLIRT,^26^, ^27^, ^28^ and $T_{1}$‐weighted images were acquired after each set of scans using a phase‐sensitive inversion recovery (PSIR) sequence^29^ (slice thickness =5mm, resolution $=1\times 1mm^{2}$, number of slices =5, FOV$=224\times 224\times 25mm^{3}$, TE=3.7ms, ${TI}_{1}=798ms$, ${TI}_{2}=2398ms$, number of echoes =1, TR=12.9ms, flip angle $=8^{\circ }$, acquisition time =40s), with the same geometry as the GE scans to allow identification and segmentation of the CC (PSIR images for all subjects are displayed in Supporting Information Figure S1).

White matter appears with a high signal intensity compared to gray matter and cerebrospinal fluid in the PSIR images, allowing the CC of each subject to be manually selected using MRIcron^30^ and then divided into 5 ROIs; the genu, anterior‐body, middle‐body, posterior‐body, and splenium as shown in Figure 6.^24^ These 5 ROIs were applied to the GE magnitude data and frequency difference maps, with signals summed and averaged over voxels in each ROI for the 6 repeats on each subject, to classify how signal magnitude and frequency difference developed with TE for individual subjects. Magnitude and frequency difference curves were additionally calculated for the superior cerebellar peduncle and a gray matter region (as shown in Figure 6).

### 3.2 Data analysis

Magnitude and frequency difference curves were simultaneously fitted to Equations 2 and 5 for the 5 ROIs in the CC (and for the superior cerebellar peduncle) of each subject to obtain values of $T_{2a,m,e}^{*}$, $A_{a,m,e}$, and $f_{a,m}$. Values of $f_{a}$ and $f_{m}$ are measured relative to $f_{e}$, which was arbitrarily set to 0*.* Fitting was performed using the “lsqnonlin” function in MATLAB (The MathWorks, Inc., Natick, MA) with the initial values and allowed ranges of the variables set as defined in Supporting Information Table S1. During fitting, residuals were scaled by the inverse standard deviation of the magnitude and frequency difference data over each ROI to account for the effective experimental SNR.

### 3.3 Practical FDM implementation

**Figure 2:**
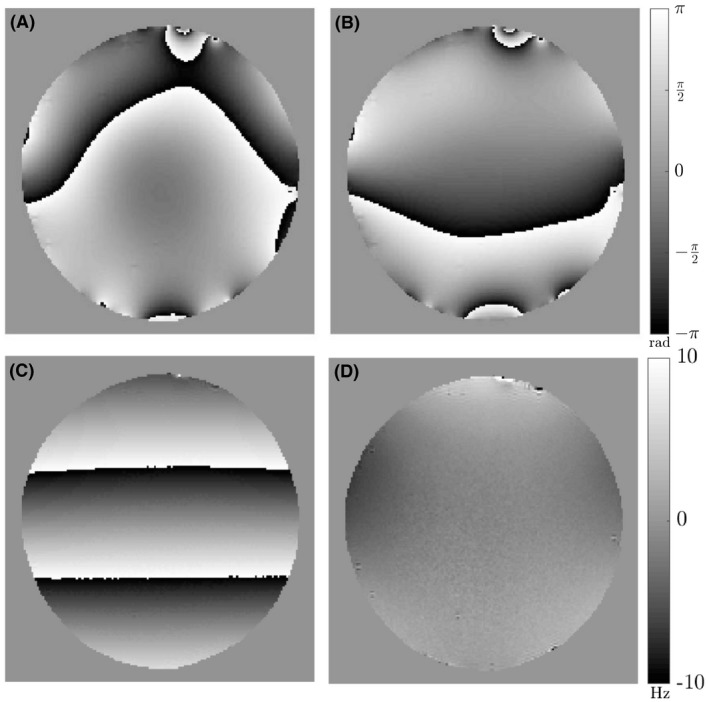
Visualization of FDM processing steps for a sagittal, single slice, multi‐echo GE scan of an agar phantom. Data shown for the sixth echo acquired at TE=14.4ms. The read gradient was applied in the foot‐head direction, which is vertical in these images. (A) Original unprocessed phase data. (B) After division by first echo data set to remove the effects of $\phi_{0}$. (C) After second scaled echo division to remove the effects of Ω. (D) Frequency difference map produced from data shown in (C) after subtraction of a 1D linear fit to the phase variation in the read direction. Phase images shown in (A–C) are scaled between ±πrad, whereas the frequency difference map shown in (D) is scaled between ±10Hz

**Figure 3:**
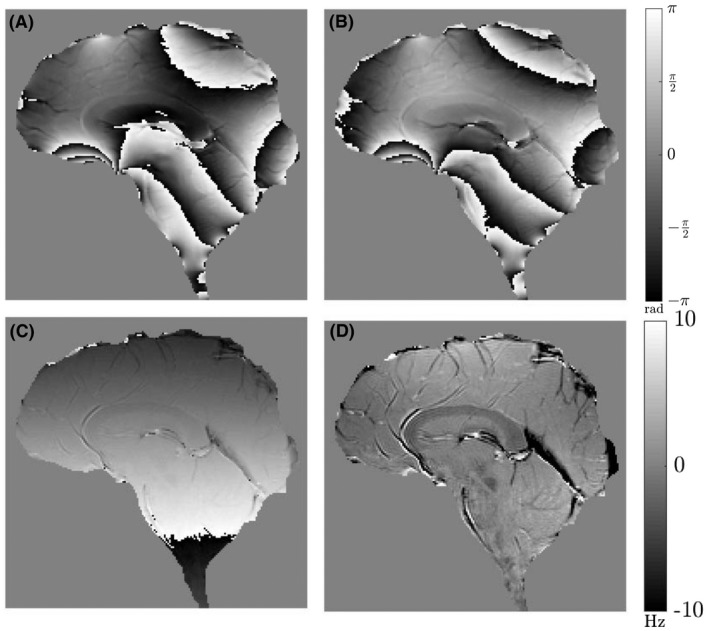
Visualization of FDM processing steps for a sagittal, single‐slice, multi‐echo GE scan acquired in vivo at the midline. Data are shown for the sixth echo acquired at TE=14.4ms. The read gradient was applied in the foot‐head direction. (A) Original unprocessed phase data. (B) After division by first echo data set to remove the effects of $\phi_{0}$. (C) After second scaled echo division to remove the effects of Ω. (D) Frequency difference map produced from data shown in (C) after subtraction of a 1D linear fit to the phase variation in the read direction. Phase images shown in (A–C) are scaled between ±πrad, whereas the frequency difference map shown in (D) is scaled between ±10Hz

We evaluated the efficacy of the FDM data analysis by applying it to data acquired from an oblate, spheroidal agar‐filled phantom (agar recipe outlined in a previous work^31^). In a homogenous phantom, the phase is expected to vary linearly with TE and the frequency difference map should therefore be null. Figure 2 shows the results of applying the FDM processing steps to the phase data from the sixth echo (TE=14.4ms). Figure 2A shows the raw phase data ($arg\left[S\left(TE_{6}\right)\right]$) in which large, length‐scale phase variation attributed to the effects of $\phi_{0}$ and Ω is evident. This variation is reduced in $arg\left[S^{\prime }\left(TE_{6}\right)\right]$ after elimination of the effect of $\phi_{0}$ by division of the complex image by the image acquired at $TE_{1}$ (Figure 2B). After elimination of the effect of Ω in calculation of $S^{\prime \prime }\left(t\right)$ using Equation 4, the phase of $S^{\prime \prime }(TE_{6}$ *)* shows an unexpected linear variation in the read gradient direction (Figure 2C). This results from small relative shifts in the sampling of the first, second, and sixth echoes introduced by the scanner’s automatic phase correction process, whose effects are amplified by raising $S^{\prime }\left(TE_{2}\right)$ to the fifth power in Equation 4. To eliminate this effect, we applied a linear and 0th‐order phase correction to $S^{\prime \prime }\left(t\right)$ separately for each value of $TE_{n}$. This was done by forming a complex image (Equation 8):

(8) $$z\left({TE}_{n}\right)=\left|S({TE}_{n})\right|\times e^{i\;\arg[S^{\prime \prime }\left(TE_{n}\right)]},$$

which was then masked and averaged in the phase encoding direction to yield a 1D profile which, after unwrapping, reveals coherent phase variation along the read direction. A first‐order polynomial was fitted to this profile, and the resulting fit was duplicated at all positions in the phase encoding direction to form a 2D image, which was subtracted from $\arg\left[S^{\prime \prime }\left(TE_{n}\right)\right]$, before calculation of the FDM using Equation 5. Figure 2D shows that the effects of the artefactual linear phase variation have been eliminated in the FDM. Figure 3 shows a similar set of data from a human subject, indicating that the processing steps also eliminate the effects of $\phi_{0}$, Ω, and artefactual linear phase variation in brain data revealing anatomical detail.

**Figure 4:**
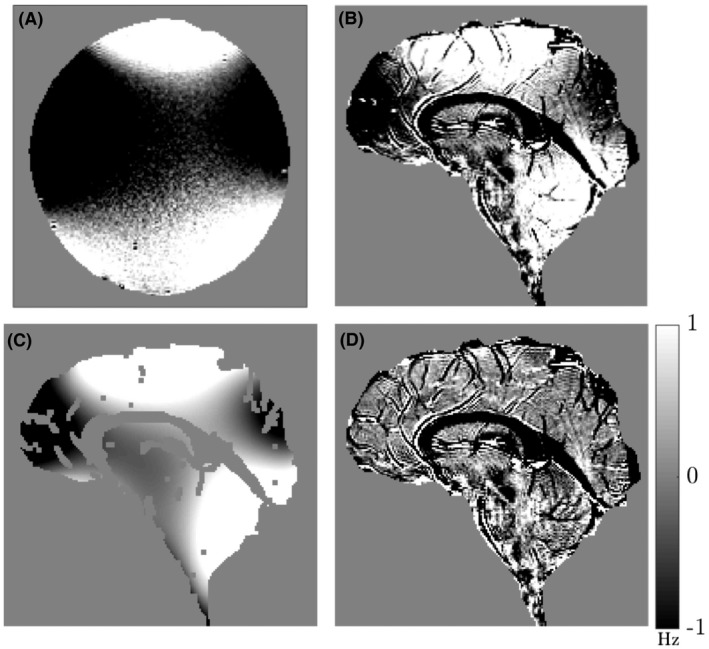
Distinctive pattern of large‐length‐scale variation of the frequency difference in maps obtained from the agar phantom (A) and head (B). Maps are shown from the sixth echo acquired at TE=14.4ms and are clipped to show values ranging between ±1Hz. (C) Sixth‐order polynomial fit to the frequency difference data (excluding white matter regions and blood vessels). (D) Frequency difference map after subtraction of the sixth‐order polynomial fit

On closer inspection, both the phantom and brain data, however, showed a distinctive pattern of large‐length‐scale variation of the frequency difference, approximately of the form $z^{2}-y^{2}$ (z is the foot‐head direction and y the anterior‐posterior direction). This is visualized in Figure 4, by windowing the FDM data shown in Figures 2D and 3D over a narrow frequency range (±1Hz). This pattern of frequency variation, which was found to be stable over repeated experiments, is most likely produced by short‐lived eddy currents which cause nonlinear phase evolution. In contrast to the frequency difference variation resulting from the anatomical distribution of myelinated structures (Figure 3D), this contribution to the frequency difference varies smoothly and relatively slowly with spatial position. It could therefore be removed from the data by subtracting a sixth‐order polynomial fit to a version of the frequency difference data in which regions of anatomical contrast (white matter and blood vessels) had been masked out. This mask was generated by thresholding the FDM image to remove all voxels with frequency values less than -3.5Hz and masking out the whole CC using the manually generated ROI as shown in Figure 4C. The sixth‐order polynomial was fit to the masked data and subsequently expanded over the entire imaging volume before subtraction from the experimental data. After subtraction of the polynomial fit, the large‐length‐scale variation is eliminated from the FDM, which consequently shows only anatomical features (Figure 4D). This fitting process was applied separately to the FDM images calculated at each TE.

## 4 RESULTS

**Figure 5:**
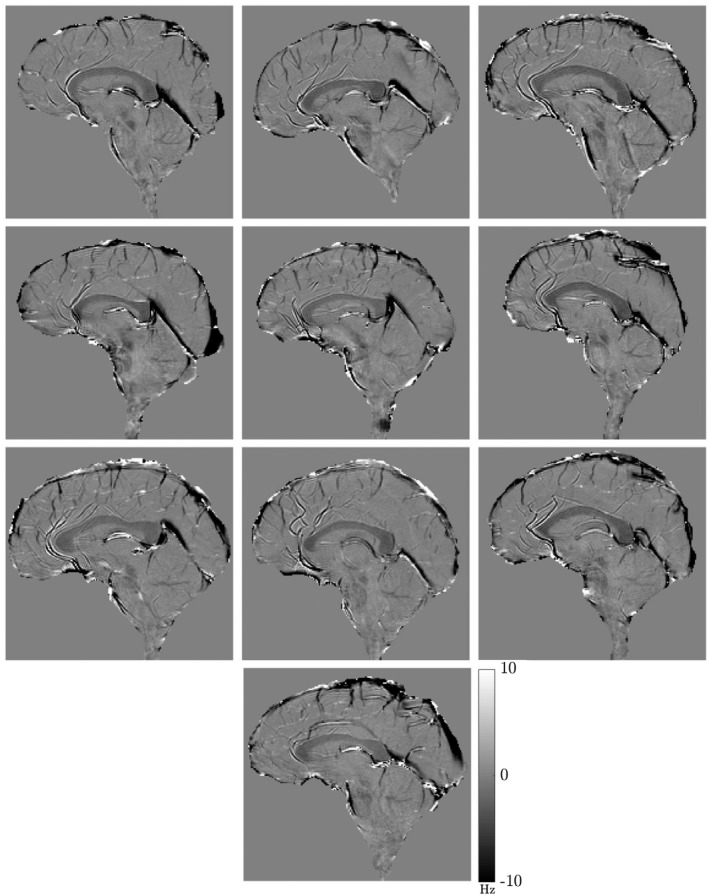
Individual frequency difference maps generated from the sixth echo acquired at TE=14.4ms for 10 subjects undergoing a single‐slice sagittal scan over the midline of the CC (parameters outlined in the Methods section). Corresponding magnitude images shown in Supporting Information Figure S2. All frequency difference maps scaled between ±10Hz

**Figure 6:**
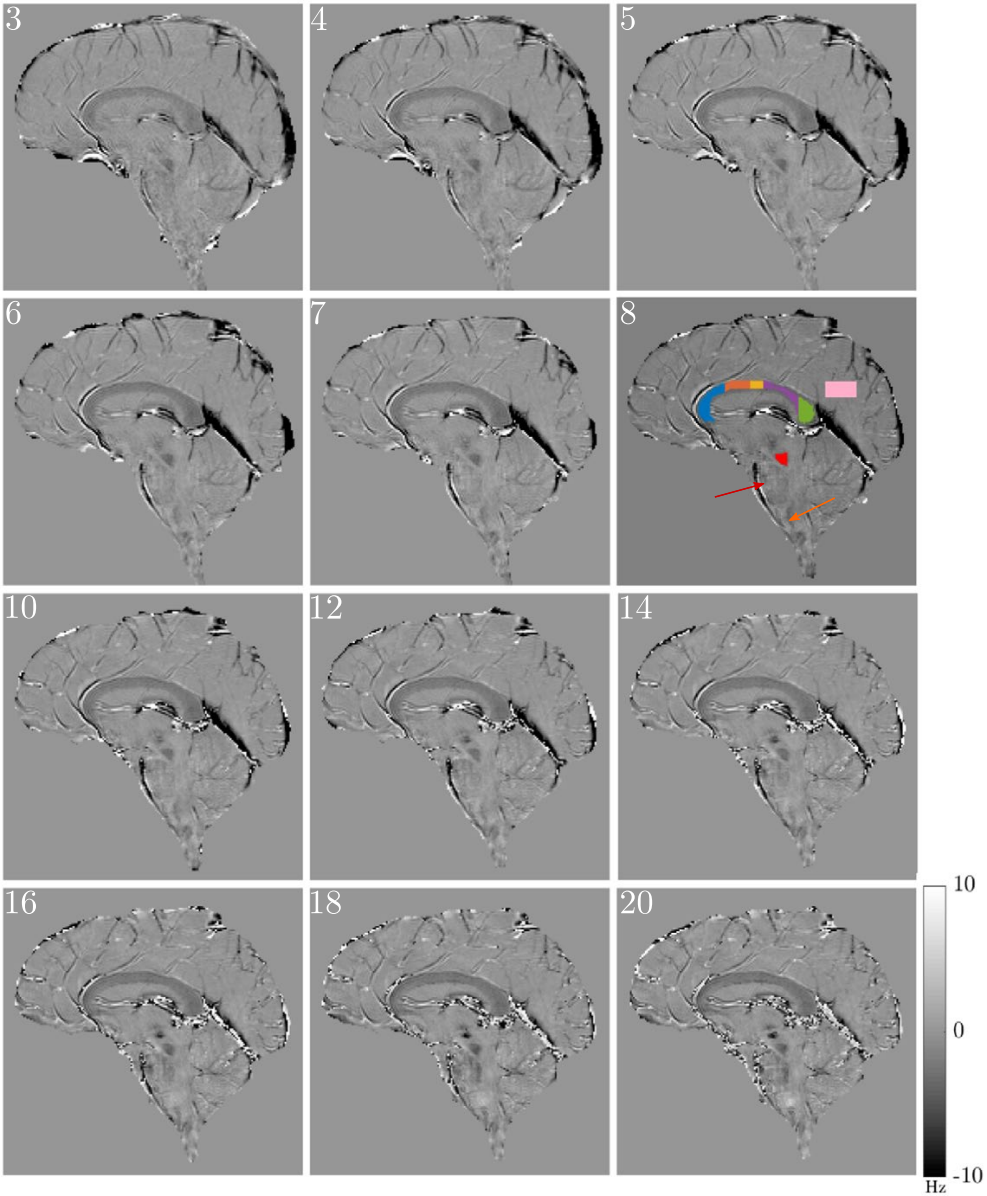
Evolution of the frequency difference with TE (echo number is indicated in the top left corner of each image) in data from a single subject starting from ${TE}_{3}=7.2ms$. ROIs are marked on the frequency difference map at ${TE}_{8}=19.2ms$. The red and yellow arrows highlight the pons and medulla oblongata, respectively. The 5 different regions of the CC used in subsequent analysis are indicated as genu (blue), anterior body (orange), middle body (yellow), posterior body (purple), and splenium (green). The red and pink shaded regions indicate the superior cerebellar peduncle and gray matter region used in further analysis. Corresponding magnitude images are shown in Supporting Information Figure S3. All frequency difference maps are scaled between ±10Hz

Frequency difference maps obtained at TE=14.4ms (sixth echo) from all 10 subjects are displayed in Figure 5. Figure 6 shows how the frequency difference maps develop with TE for a single subject (corresponding magnitude data are shown in Supporting Information Figures S2 and S3). Note that in Figure 6 (and subsequent figures showing FDM data), the range of TE values shown starts at the third echo (TE=7.2ms) because the FDM processing does not yield a value at the first echo (Equation 3) and forces the value to 0 at the second echo (Equation 4). The effects of $\phi_{0}$ and Ω have evidently been removed in the FDM‐processed images and anatomical structure is consistently depicted in these frequency difference maps, with white matter and blood vessels providing the dominant source of contrast. The CC is clearly depicted as hypointense compared to the surrounding gray matter, with the signal becoming more hypointense with increasing TE at the early TEs. Other structures, such as the superior cerebellar peduncle, the pons, and medulla oblongata (see Figure 6), which contain white matter fiber bundles running perpendicular to B_0_, are also visible in the frequency difference maps.

**Figure 7:**
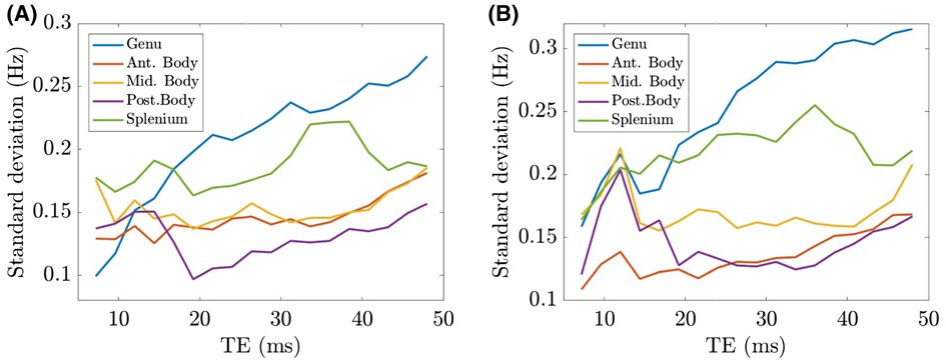
Variation of the standard deviation of the frequency difference values within the 5 different ROIs in the corpus callosum. (A) was formed by calculating the average standard deviation of the mean value within the ROI for each set of 3 repeats within the scanner across all 10 subjects. (B) was formed by calculating the average of the standard deviation of the mean value within the ROI for the 2 scan sets (3 scans per set, 2 sets per subject) across all 10 subjects

Variation with TE of the standard deviation of the measured frequency difference within the 5 different ROIs in the CC is shown in Figure 7: Figure 7A shows the average standard deviation over the sets of 3 measurements made without moving the subject out of the scanner, whereas Figure 7B shows the standard deviation between scanning sessions (the subject is repositioned in the scanner between scanning session). Figure 7A was formed by calculating the average standard deviation across all 10 subjects of the mean value within the ROI for each set of 3 repeated acquisitions made without moving the subject out of the scanner; Figure 7B was formed by calculating the average of the standard deviation of the mean value within the ROI for the 2 scan sets (3 scans per set, 2 sets per subject) across all 10 subjects. In both cases, the standard deviation increases with TE and is largest in the genu, but has a maximum value which is <0.32Hz. A voxel‐wise standard deviation map for a single subject is shown in Supporting Information Figure S4.

**Figure 8:**
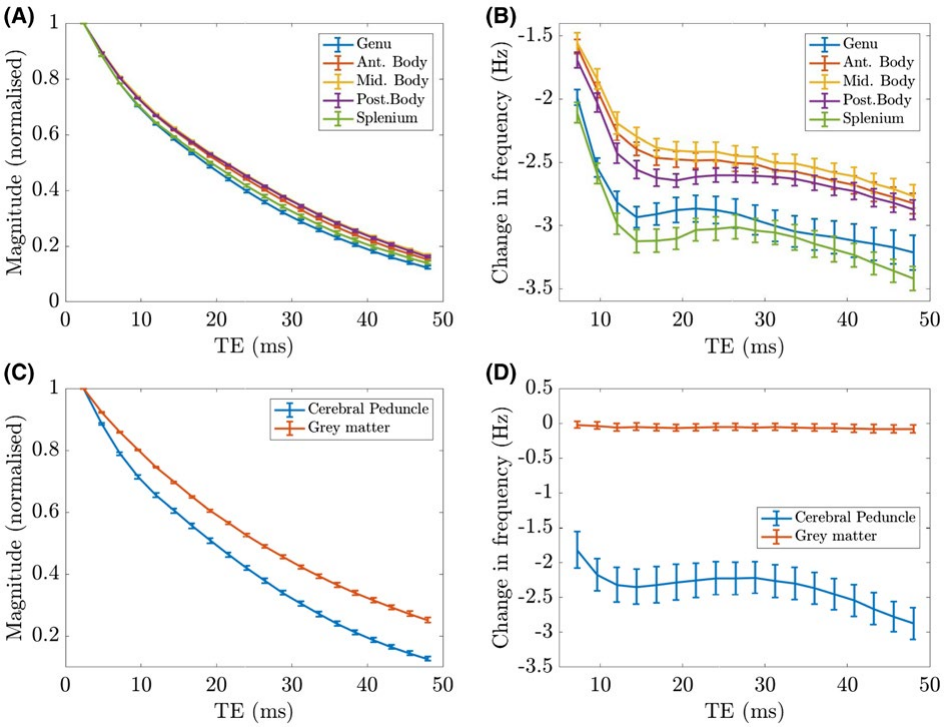
Variation of the average magnitude (A,C) and frequency difference (B,D) values with TE for the 5 different ROIs in the CC (A,B), superior cerebellar peduncle, and gray matter region (C,D). These are formed from the average of 60 scans acquired from 10 different subjects. Error bars represent the average standard error from each set of 6 repeats per subject. Note that the frequency difference values are only shown for TE≥7.2ms, because the frequency difference is not defined at $TE_{1}$ and forced to 0 at $TE_{2}$

Figure 8A and B shows the variation of the mean magnitude and frequency difference with TE in the 5 ROIs of the CC. These curves were formed by averaging over the data sets from individual subjects shown in Supporting Information Figure S5. All regions show a negative frequency difference, which increases in magnitude significantly over the first 3 to 6 TEs (TE=7.2-14.4ms) and then displays smaller variations over the subsequent echoes (this motivated the selection of the sixth echo for visualizing the frequency difference maps in other figures, as the frequency difference values plateaued around the sixth echo time). The characteristic magnitude of the frequency differences at the later TEs is 2.5to3.5Hz, which is approximately 10 times larger than the largest standard deviation reported in Figure 7. The genu and splenium display a more‐rapid magnitude decay compared to the central regions of the CC, along with a larger frequency difference.

Figure 8C and D shows the average across all subjects of the magnitude and frequency difference in the superior cerebellar peduncle and a region of grey matter (ROIs for both regions are highlighted in Figure 6). Little change in frequency with TE is observed for the gray matter ROI in Figure 8D, whereas the superior cerebellar peduncle, which contains myelinated nerve fibers oriented perpendicular to $B_{0}$, shows a frequency difference similar to that measured in the CC. The rate of decay of the magnitude signal from the superior cerebellar peduncle is similar to that observed in the CC, and, as expected, the rate of signal decay is lower in the gray matter region, attributable to the higher $T_{2}^{*}$ of gray matter.^1^

**Figure 9:**
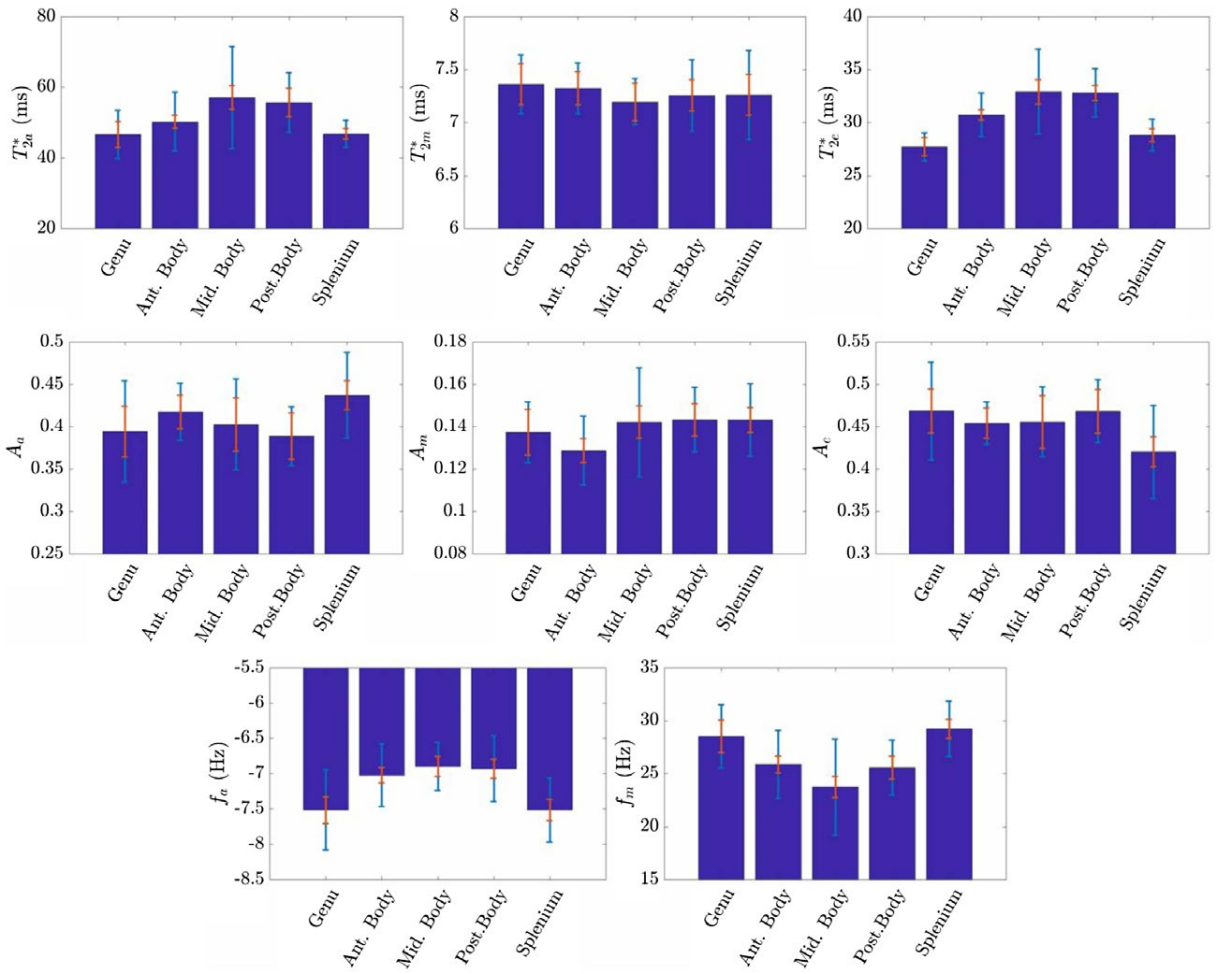
Average values of of $T_{2a,m,e}^{*}$, $A_{a,m,e}$, and $f_{a,m}$ produced by fitting the magnitude of Equations 2 and 5 to the experimentally measured magnitude and FDM curves from 10 subjects. Here, the orange line represents the average standard error from each set of 6 repeats per subject, and the light blue line represents the standard deviation between subjects derived from the mean per‐subject parameter value

Figure 9 displays the variation over the 5 ROIs in the CC of the parameters ($A_{a,m,e}$, $T_{2a,m,e}^{*}$, and $f_{a,m}$) derived from fitting the magnitude of Equations 2 and 5 to the measured magnitude and FDM curves. The fitted parameters for the superior cerebellar peduncle are shown in Supporting Information Table S2. Supporting Information Figure S6 displays the average residual remaining after subtracting the model fit from the measured magnitude and FDM data. The average residuals are less than 1% and 0.4Hz for the magnitude and FDM data, respectively.

**Figure 10:**
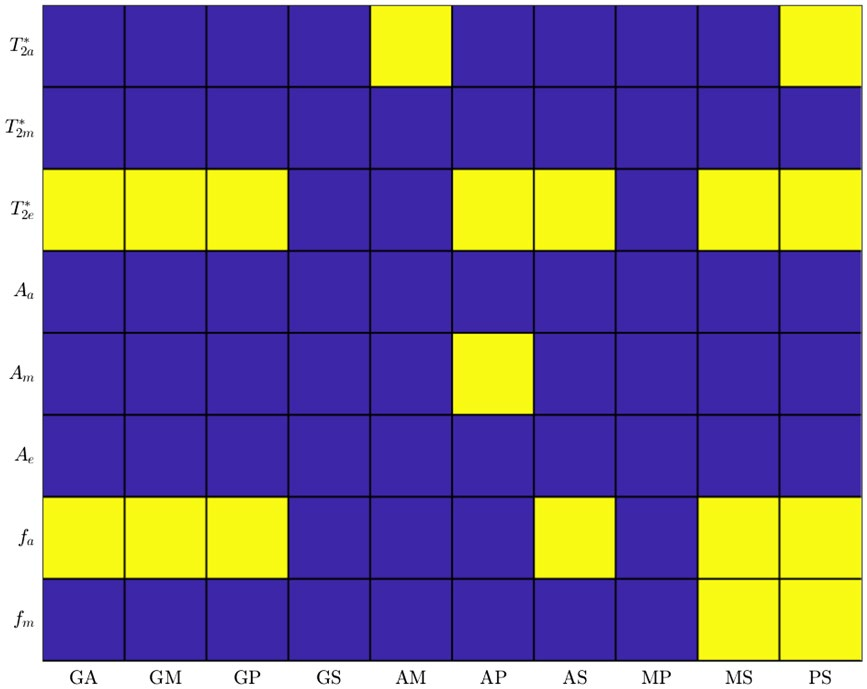
Results for Wilcoxon signed‐rank test comparing different regions for the fitted and calculated parameters. Here, G = genu, A = anterior body, M = middle body, P = posterior body, and S = splenium. The x‐axis represents the 2 regions being compared (e.g., GA compares the genu and anterior body). Yellow cells represent the rejection of the null hypothesis at P≤0.05; in blue cells, the null hypothesis is not rejected. A Bonferroni correction has been applied to account for the multiple parameters compared between pairs of ROI

Figure 10 schematically displays the results for the Wilcoxon signed‐rank test of the significance of the differences in the fitted and calculated parameters between ROIs. In the case of the fitted parameters, there are significant differences between the values found in the splenium/genu and those in the anterior/middle/posterior bodies (most consistently in $T_{2e}^{*}$ and $f_{a}$) and some differences between the anterior body and the middle/posterior bodies, reflecting the pattern of differences in the data shown in Figure 9.

## 5 DISCUSSION

The relatively simple processing pipeline for multi‐echo GE data that we have developed allows the effective elimination of phase variation due to RF phase effects ($\phi_{0}$), as well as removing the effects of the average frequency offset in each voxel (Ω). The latter includes the effects of sources of field variation located outside the brain, which can be eliminated by background field removal procedures,^32^ as well as field offsets produced by the voxel‐scale variation of magnetic susceptibility in the brain, which is exploited in susceptibility mapping. The resulting frequency difference maps consistently show clear contrast between the CC and the surrounding gray matter, as well as highlighting the superior cerebellar peduncle, which is another white matter structure in which the nerve fibers are oriented perpendicular to $B_{0}$ (Figure 5). These white matter regions show negative contrast, mainly because of the rapid decay of the signal from the myelin water pool, which shows a positive frequency offset relative to the signals from the axonal and external compartments. Individual frequency difference maps may therefore be used to provide a qualitative indication of the presence of myelinated nerve fibers, and potentially thus to identify areas of demyelination in multiple sclerosis. Although the frequency difference maps generated here were based on single‐slice images, the FDM approach can equally be applied to multislice and 3D data.^7^, ^9^, ^23^

Here, we acquired echo trains containing 20 gradient echoes, with TEs that were multiples of 2.4ms, but Figures 6 and 8 show that the frequency difference contrast is already close to the maximum value attained at the sixth echo (TE=14.4ms), so useful frequency difference maps could be obtained with echo trains of significantly shorter duration than the ~50ms used here.

Small veins also appear with low intensity in the frequency difference maps, as a result of the frequency offset and short relaxation time of the venous blood compared to the surrounding tissue. Arteries also produce adjacent bright and dark regions in the frequency difference maps. This is most likely a consequence of flow effects. Flow compensation was not used here, to limit the first TE and the echo spacing, and because phase encoding is applied before the start of the echo train, the position that flowing blood will appear in the image in the anteroposterior direction is the same for images generated at all TEs, whereas its position in the foot‐head, read direction changes down the echo train if the flow is such that the blood is displaced along this direction. This effect is particularly evident where the anterior cerebral artery curves round the genu of the CC (Figures 5 and 6). It may be possible to reduce these effects by applying flow compensation to the read gradient waveform, and rewinding and reapplying the phase encoding after each echo. However, this would increase the TEs, which would reduce sensitivity to the myelin water signal. The processing pipeline used here to generate frequency difference maps also includes steps needed to eliminate small differences in the echo position relative to the sampling window that were introduced by the scanner’s automatic phase correction process (see Figures 2C and 3C), and to eliminate residual large‐length‐scale variation of the frequency difference, which is most likely caused by short‐lived eddy currents. Disabling the scanner’s automatic phase correction allows the first step to be omitted, but further investigation is needed to identify the origin of the short‐lived eddy currents and establish whether these effects can be eliminated at source or at least reduced from the ~1-Hz amplitude they take in our data.

The evolution of the frequency difference with TE in ROIs in the CC and superior cerebellar peduncle (Figure 8) shows a characteristic reduction at early TEs attributed to loss of the signal from the myelin compartment, whereas the variation at later TEs reflects the interference between the complex signals from the external and axonal compartments, resulting from the negative axonal frequency offset. The evolution of the frequency difference in the gray matter ROI, shown in Figure 8, does not show significant variation with TE as expected. This ROI was positioned in a region of relatively uniform signal in the PSIR images, and given the positioning of the 5-mm-thick sagittal slice at the midline and the appearance of the tissue in the PSIR image, it is expected mainly to contain gray matter. It is possible that because of the cortical folding, there is some partial voluming with the white matter that is proximal to the cortical layer. However, given that it is likely that there will be a range of fiber orientations in such white matter, we do not expect such partial voluming to produce a significant effect on the frequency difference values.

The characteristic size of the frequency difference in the CC is 2 to 3Hz in these data, where the first TE and echo spacing were both 2.4ms. Changing these TEs will have some effect on the absolute frequency difference values. Figure 7 shows that the standard deviation of the frequency difference values in the ROIs within the CC generally increases with TE, but is always less than 0.32Hz. The largest values of standard deviation are noted in the genu and splenium. Overall, these standard deviations, which were assessed experimentally by repeatedly scanning each subject, provide some confidence in the possibility of characterizing differences in the frequency difference values at the sub‐Hz. The scaling of the standard deviation of the frequency difference in the ROI with echo number does not follow the theoretical form shown in Figure 1 that is based on simple propagation of white noise in the real and imaginary signals. This implies that other sources of phase noise, such as field variation caused by respiration and other physiological effects also need to be considered. The first‐echo magnitude SNR of 300 used in Figure 1E reflects the average voxel SNR value found in the experimental data.

Figure 8 reveals an ordered variation of signal behavior across the CC, with the splenium and genu displaying faster decay of the signal magnitude and a larger change in frequency with TE than the midregions of the CC. This ordering of the magnitude and frequency difference curves across the CC was also observed consistently in the measurements from the individual subjects (see Supporting Information Figure S4). The frequency difference curves from the splenium and genu lie below those from the midregions of the CC for all relevant TEs in 5 of the 10 subjects, and for the majority of the TEs in most of the subject data. In all but 1 of the subjects, the frequency difference values were more negative in the splenium and genu at the first 2 accessible TE values (7.2 and 9.6ms). The magnitude signals from the genu and splenium decay faster than those from the midregions of the CC in 8 of the 10 subjects (in the other subjects, the normalized signal from the splenium exceeded that from the anterior body at the late TEs).

The parameters produced by fitting the three‐pool model to the FDM and magnitude data (Figure 9) conform to the expected behavior, with the myelin water signal having a considerably shorter relaxation time (7.2–7.4ms) than that found in the axonal (46.6–57.0ms) and external (27.7–32.9ms) compartments and a smaller signal amplitude, which is around 14% of the total signal. In addition, as predicted by the hollow cylinder model, the average frequency offset in the myelin compartment is positive with respect to that in the external compartment, ranging from 23.7–29.3Hz, whereas the frequency offset in the axonal compartment is negative and smaller in amplitude (-7.6to-6.8Hz). The larger FDM and faster rate of signal decay in the genu and splenium compared to the other regions of the CC are most obviously manifested in the fitted parameters in the greater magnitudes of $f_{a}$ and $f_{m}$ and the reduced values of $T_{2a}^{*}$ and $T_{2e}^{*}$ in the splenium and genu, although these differences are not all statistically significant (Figure 10). The amplitudes of the signals from the different compartments, $A_{a,m,e}$, do not show a clear pattern of variation across the different regions of the CC (Figure 9), and the only significant difference found by the Wilcoxon signed‐rank test is the lower value of $A_{m}$ in the anterior body compared to the posterior body. The values of the fitted parameters for the splenium are in excellent agreement with data previously reported by Sati et al (who analyzed complex multi‐echo gradient echo signals acquired from the splenium at 7T): Our fitted parameter values fall within 2 standard deviations of Sati et al’s results.^8^ The correspondence of our measurements with the values recently reported by Thapaliya et al, who analyzed complex gradient signals measured from similar ROIs in the CC at 7T,^20^ is less clear. The frequency offsets in the axonal and myelin compartments that these researchers report are similar in magnitude to our values, but the relaxation times in the axonal compartment are significantly larger in their data. They also found that the signal contribution from the axonal compartment was significantly larger than that from the external compartment, whereas we found that $A_{a}$ and $A_{e}$ were approximately equal in magnitude. These discrepancies may be explained by differences in the approach to data analysis. Thapaliya et al used iHARPERELLA^34^ to unwrap the phase and remove the background field effects from images acquired at each TE—an approach which has been shown to produce some differences in phase evolution with TE compared with path‐based unwrapping approaches.^18^ In addition, they did not consider the effect of spatially varying, time‐independent phase (i.e., $\phi_{0}$) in their fitting, and their ROI‐based analysis involved fitting to the sum of the complex signal over all voxels in the ROI. Phase variation across the ROI resulting from the fields produced by the tissues of the brain (i.e., the variation that remains after background field removal) will cause additional complex signal variation with TE, which may influence the fit to the three‐pool model. Our data may have suffered to some extent from this effect because of the use of 5-mm slice thickness, which could have given rise to through‐slice TE‐dependent dephasing.

In this work, we focused on white matter regions (CC and superior cerebellar peduncle) in which the effects of microstructure on the GE signals are greatest because the nerve fibers are coherently oriented perpendicular to $B_{0}$. Previous work has shown that there are measurable, but lesser, frequency differences in fiber tracts with other orientations with respect to $B_{0}$ and that these can be explained by using a simple hollow cylinder model of the nerve fibers, in which the frequency offsets of the intra‐axonal and myelin water signals depend on the angle that the fibers make to the field.^7^, ^8^, ^9^ This model also explains why the rate of decay of the GE signal depends on fiber orientation, given that the magnitude of the spatially varying fields produced by the myelin sheath varies with the angle that the fibers make to the field.^7^, ^8^ The orientation dependence of the frequency differences and relaxation rates introduces an additional complication in relating microstructure to signal characteristics, but the three‐pool model can still generally be used to characterize the relative magnitudes of the signals from the axonal, myelin, and external compartments.^16^ In addition, it is possible to use diffusion tensor imaging to estimate fiber orientations with respect to the field, and then to use this information to improve the modeling of the evolution of GE signals.^9^ The effect of fiber orientation dispersion should also be considered in future work. As a result of the orientation dependence of the frequency offsets, such dispersion is likely to produce accelerated signal decay because of the increased range of frequencies appearing in the axonal and myelin water signals.

## 6 CONCLUSION

The FDM approach that we have described allows straightforward generation of images which show contrast in areas where the phase of multi‐echo GE signals evolves nonlinearly. Nonlinear phase evolution occurs in the presence of tissue microstructure, such that voxels contain multiple compartments, with different compartments producing NMR signals that evolve at different frequencies and decay at different rates. Myelinated nerve fibers in white matter and small venous blood vessels are the dominant source of contrast in frequency difference maps of the brain. FDM consequently provides a simple way of producing image contrast that highlights voxels containing coherently ordered myelinated nerve fibers, and can therefore also potentially be used to highlight areas of demyelination (e.g., in multiple sclerosis lesions).^33^ Here, we showed that FDM shows strong contrast in the CC and superior cerebellar peduncle in sagittal maps acquired at the midline. By analyzing the evolution of the frequency difference and signal magnitude in 5 regions of the CC, we found that the frequency difference was significantly larger, and the rate of magnitude signal decay was also greater in the splenium and genu, than in the midregions of the CC. Fitting these data to a three‐pool model indicated that these differences mainly resulted from differences in the $T_{2}^{*}$ relaxation rates in the axonal and external compartments and the frequency offsets in the axonal and myelin compartments.

## Supporting information


**FIGURE S1** PSIR images from the 10 subjects over the midline of the CC (parameters described in the Methods section)
**FIGURE S2** Magnitude images generated from the sixth echo acquired at TE=14.4ms from the 10 subjects undergoing a single‐slice sagittal scan over the midline of the CC (parameters described in the Methods section). Corresponding frequency difference maps are shown in Figure 5 of the main text. All echoes are normalized to first echo image (TE=2.4ms) to show relative signal amplitude
**FIGURE S3** Evolution of the signal magnitude with echo time in data from a single subject starting from ${TE}_{3}=7.2ms$. Corresponding frequency difference maps are shown in Figure 6 of the main text. All echoes are normalized to the first echo image (TE=2.4ms) to show relative signal amplitude
**FIGURE S4** Comparison of the frequency difference map for a single scan (a) and a map of the standard deviation (b) of the frequency difference over 3 repeats of the acquisition on the same subject in a single scanning session (sixth echo, TE=14.4ms). The pink line outlines the CC region selected for analysis
**FIGURE S5** Magnitude and frequency difference measured from the 5 ROIs in the CC for the 10 individual subjects. Each pair of plots is averaged over the 6 repeats per subject, with error bars representing the standard error over those repeats
**FIGURE S6** Variation with TE of the average residuals of the magnitude (a) and frequency difference (b) data after subtraction of the model fits. Residuals were calculated from each individual data set (6 per subject for 10 subjects) with errors formed from the average standard error from each set of 6 repeats per subject
**TABLE S1** Parameter values (initial and range) used in fitting experimental data. Initial and min/max values of amplitudes $A_{a,m,e}$ were chosen based on the maximal allowed range of these parameters. For $f_{a,m}$, the min/max values were chosen as sensible limits for the maximum deviation from 0Hz, well below previous estimates^2^, ^3^ of $f_{a,m}$. Unlike the initial values of $A_{a,m,e}$ and $f_{a,m}$, the initial values of $T_{2a,m,e}^{*}$ were defined based on previous literature estimates,^2^, ^3^, ^4^ to ensure that fitting would define each compartment as corresponding consistently to a short, medium, and long $T_{2}^{*}$ pool.
**TABLE S2** Average values of of $T_{2a,m,e}^{*}$, $A_{a,m,e}$, and $f_{a,m}$ produced by fitting the magnitude of Equation 2 (main text) and Equation 5 (main text) to the experimentally measured magnitude and FDM curves from the superior cerebellar peduncle in 10 subjects, with the average standard error from each set of 6 repeats per subject and the standard deviation between subjects derived from the mean per‐subject parameter value Note that in 9 of the 60 data sets, the value of $T_{2a}^{*}$ (3 occurrences) or $T_{2e}^{*}$ (6 occurrences) converged on the upper fitting boundary of 100ms.
