# Supplementary material for: Frequency difference mapping applied to the corpus callosum at 7T
Source: Magn Reson Med. 2018 Dec 23;81(5):3017–31. doi: 10.1002/mrm.27626 (PMC6492142; doi:10.1002/mrm.27626)
Supplement: Supplementary file 1 — FIGURE S1 PSIR images from the 10 subjects over the midline of the CC (parameters described in the Methods section) FIGURE S2 Magnitude images generated from the sixth echo acquired at TE=14.4ms from the 10 subjects undergoing a single‐slice sagittal scan over the midline of the CC (parameters described in the Methods section). Corresponding frequency difference maps are shown in Figure 5 of the main text. All echoes are normalized to first echo image (TE=2.4ms) to show relative signal amplitude FIGURE S3 Evolution of the signal magnitude with echo time in data from a single subject starting from TE3=7.2ms. Corresponding frequency difference maps are shown in Figure 6 of the main text. All echoes are normalized to the first echo image (TE=2.4ms) to show relative signal amplitude FIGURE S4 Comparison of the frequency difference map for a single scan (a) and a map of the standard deviation (b) of the frequency difference over 3 repeats of the acquisition on the same subject in a single scanning session (sixth echo, TE=14.4ms). The pink line outlines the CC region selected for analysis FIGURE S5 Magnitude and frequency difference measured from the 5 ROIs in the CC for the 10 individual subjects. Each pair of plots is averaged over the 6 repeats per subject, with error bars representing the standard error over those repeats FIGURE S6 Variation with TE of the average residuals of the magnitude (a) and frequency difference (b) data after subtraction of the model fits. Residuals were calculated from each individual data set (6 per subject for 10 subjects) with errors formed from the average standard error from each set of 6 repeats per subject TABLE S1 Parameter values (initial and range) used in fitting experimental data. Initial and min/max values of amplitudes Aa,m,e were chosen based on the maximal allowed range of these parameters. For fa,m, the min/max values were chosen as sensible limits for the maximum deviation from 0Hz, well below previous estimates2, 3 of fa,m. [file MRM-81-3017-s001.docx]

**Supporting Information**

**Noise propagation in FDM calculation**

We calculate the frequency difference using:

$\mathrm{FDM}\left( TE_{n} \right)=\frac{\arg\left[ S^{''}\left( TE_{n} \right) \right]}{2\pi\left( TE_{n}-TE_{2} \right)}=\frac{1}{2\pi\left( TE_{n}-TE_{2} \right)}\left( \arg\left[ S\left( TE_{n} \right) \right]+\left( n-2 \right) \arg\left[ S\left( TE_{1} \right) \right]-\left( n-1 \right)\arg\left[ S\left( TE_{2} \right) \right] \right)$ [S1]

and since for a complex variable, $Z$, the standard deviation of $\arg\left[ Z \right]$ is approximately equal to the inverse of the signal-to-noise ratio (SNR) of $\left| Z \right|,$ which is the standard deviation of $\left| Z \right|$ divided by the average value of $|Z|$ for SNR >> 1 (1), we find that:

$\mathrm{noise}\left( TE_{n} \right)=\frac{1}{2\pi\left( TE_{n}-TE_{2} \right)}\sqrt{\left( \frac{1}{\mathrm{SNR} \left( TE_{n} \right)} \right)^{2}+\left[ \left( \frac{n-2}{\mathrm{SNR} \left( TE_{1} \right)} \right)^{2} \right]+\left[ \left( \frac{n-1}{\mathrm{SNR} \left( TE_{2} \right)} \right)^{2} \right]}$. [S2]

If we assume that the signal magnitude scales with TE as $e^{-R_{2}^{*}\mathrm{TE}}$, while the noise in the magnitude signal is independent of TE, then since ${\mathrm{TE}_{n}=TE}_{1}+\left( n-1 \right)\Delta\mathrm{TE}$:

$\mathrm{SNR} \left( TE_{n} \right)= e^{-R_{2}^{*}\left( n-1 \right)\Delta\mathrm{TE}}\mathrm{SNR} \left( TE_{1} \right)$, [S3]

and hence:

$\mathrm{noise}\left( TE_{n} \right)=\frac{1}{2\pi\left( n-2 \right)\Delta\mathrm{TE}\mathrm{SNR} \left( TE_{1} \right)}\sqrt{\left( e^{R_{2}^{*}\left( n-1 \right)\Delta\mathrm{TE}} \right)^{2}+\left( n-2 \right)^{2}+\left( \left( n-1 \right)e^{R_{2}^{*}\Delta\mathrm{TE}} \right)^{2}}$, [S4]

which can be simplified to give the expression:

$\mathrm{noise}\left( \mathrm{TE}_{n} \right)= \left( 2\pi\Delta TE \mathrm{SNR}_{1} \right)^{-1}\sqrt{\left( \frac{e^{\left( n-1 \right)R_{2}^{*}\Delta TE}}{n-2} \right)^{2}+1+\left( \frac{\left( n-1 \right)e^{R_{2}^{*}\Delta TE}}{n-2} \right)^{2}}$. [S5]

**
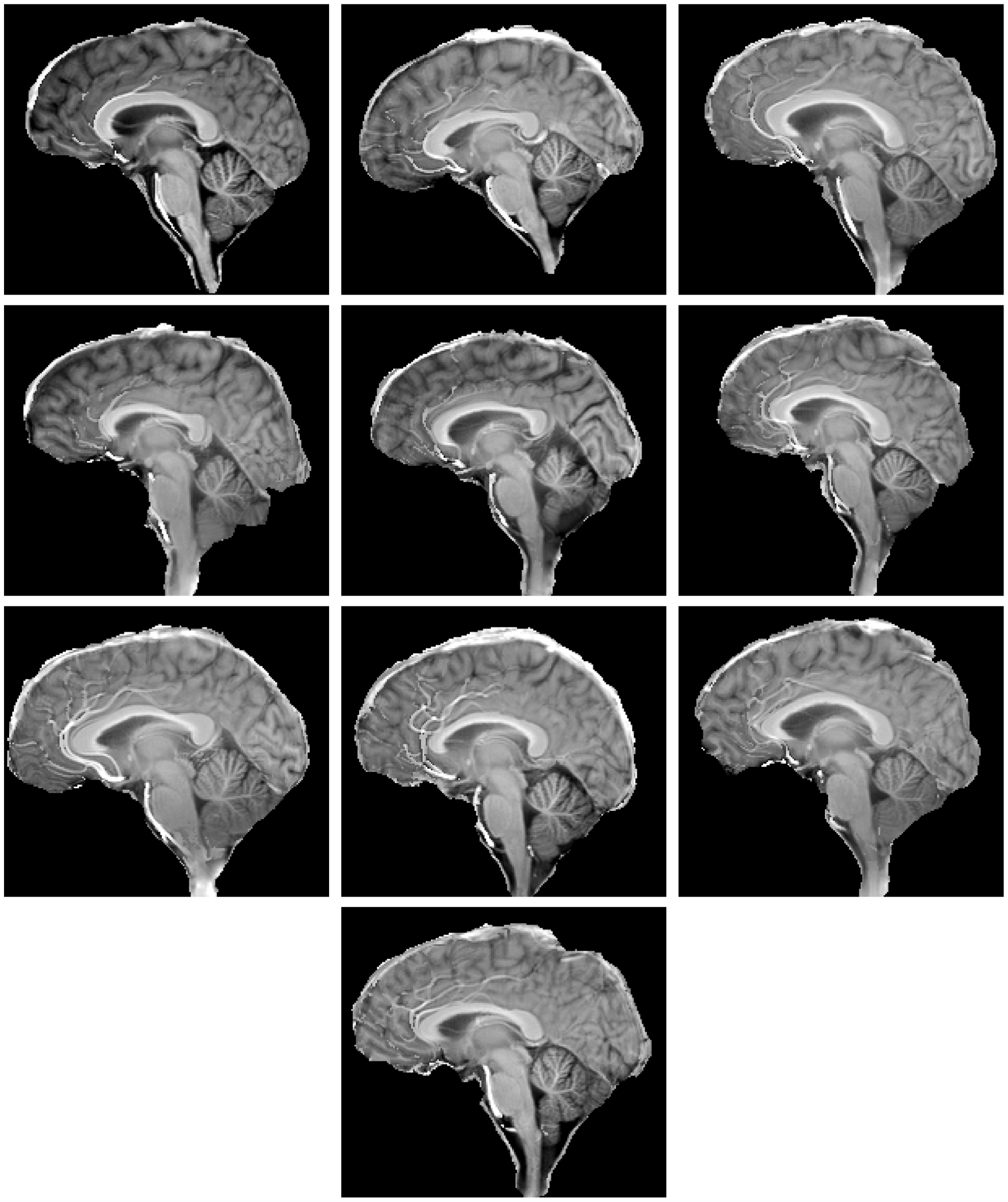
**

**Figure S1**: PSIR images from the 10 subjects over the midline of the corpus callosum (parameters described in Methods section).


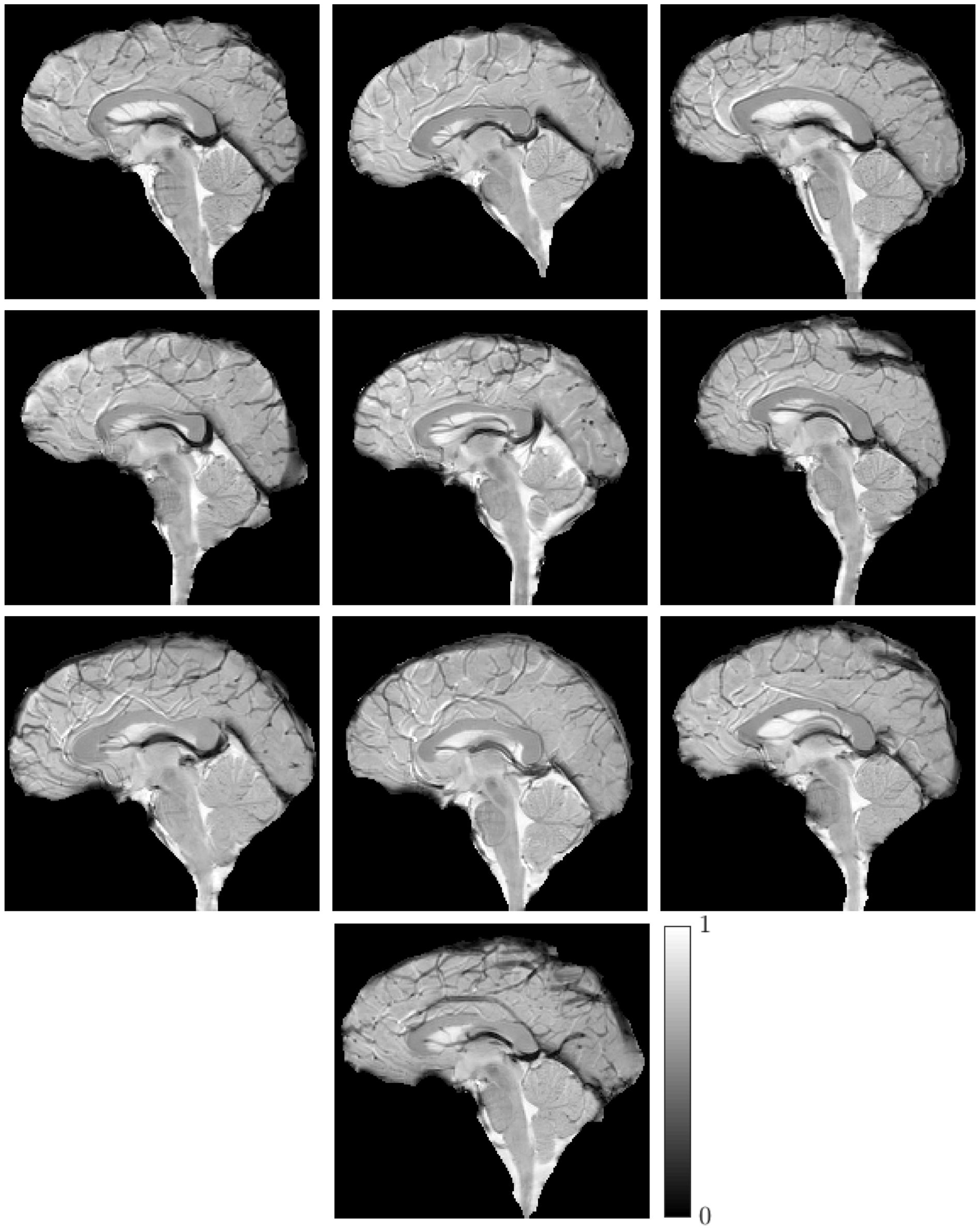


**Figure S2**: Magnitude images generated from the 6^th^ echo acquired at $TE=14.4 ms$ from the 10 subjects undergoing a single slice sagittal scan over the midline of the corpus callosum (parameters described in Methods section). Corresponding frequency difference maps are shown in Figure 5 of the main text. All echoes are normalised to first echo image ($TE=2.4 ms$) to show relative signal amplitude.

**Figure S3**: Evolution of the signal magnitude with echo time in data from a single subject starting from $\mathrm{TE}_{3}=7.2 ms$. Corresponding frequency difference maps are shown in Figure 6 of the main text. All echoes are normalised to the first echo image ($TE=2.4 ms$) to show relative signal amplitude.

**Figure S4**: Comparison of the frequency difference map for a single scan (a) and a map of the standard deviation (b) of the frequency difference over 3 repeats of the acquisition on the same subject in a single scanning session (6^th^ echo, $TE=14.4 ms$). The pink line outlines the corpus callosum region selected for analysis.

**Figure S5**: Magnitude and frequency difference measured from the 5 ROI in the corpus callosum for the ten individual subjects. Each pair of plots is averaged over the six repeats per subject, with error bars representing the standard error over those repeats.

Figure S6: Variation with TE of the average residuals of the magnitude (a) and frequency difference (b) data after subtraction of the model fits. Residuals were calculated from each individual data set (6 per subject for 10 subjects) with errors formed from the average standard error from each set of six repeats per subject.

| Parameter | $T_{2a}^{*}(ms)$ | $T_{2m}^{*}(ms)$ | $T_{2e}^{*}(ms)$ | $A_{a}$ | $A_{m}$ | $A_{e}$ | $f_{a}(Hz)$ | $f_{m}(Hz)$ |
| --- | --- | --- | --- | --- | --- | --- | --- | --- |
| Init. value | $40$ | $8$ | $25$ | $0.5$ | $0.5$ | $0.5$ | $0$ | $0$ |
| Min. value | $0$ | $0$ | $0$ | $0$ | $0$ | $0$ | $-100$ | $-100$ |
| Max. value | $100$ | $100$ | $100$ | $1$ | $1$ | $1$ | $100$ | $100$ |

**Table S1**: Parameter values (initial and range) used in fitting experimental data. Initial and min/max values of amplitudes $A_{a,m,e}$ were chosen based on the maximal allowed range of these parameters. For $f_{a,m}$, the min/max values were chosen as sensible limits for the maximum deviation from $0 \mathrm{Hz}$, well below previous estimates (2,3) of $f_{a,m}$*.* Unlike the initial values of $A_{a,m,e}$ and $f_{a,m}$, the initial values of $T_{2a,m,e}^{*}$ were defined based on previous literature estimates (2–4), to ensure that fitting would define each compartment as corresponding consistently to a short, medium and long $T_{2}^{*}$ pool.

| Parameter | $T_{2a}^{*}(ms)$ | $T_{2m}^{*}(ms)$ | $T_{2e}^{*}(ms)$ | $A_{a}$ | $A_{m}$ | $A_{e}$ | $f_{a}(Hz)$ | $f_{m}(Hz)$ |
| --- | --- | --- | --- | --- | --- | --- | --- | --- |
| Mean | 50.2 | 7.4 | 40.2 | 0.46 | 0.14 | 0.41 | -8.2 | 29.9 |
| Standard error | 8.4 | 1.2 | 11.0 | 0.08 | 0.03 | 0.08 | 0.6 | 4.5 |
| Standard deviation | 8.6 | 1.1 | 6.0 | 0.07 | 0.03 | 0.06 | 0.8 | 3.4 |

**Table S2**: Average values of of $T_{2a,m,e}^{*}$, $A_{a,m,e}$, and $f_{a,m}$ produced by fitting the magnitude of Eq. [2] (main text) and Eq. [5] (main text) to the experimentally measured magnitude and FDM curves from the superior cerebellar peduncle in 10 subjects, with the average standard error from each set of six repeats per subject and the standard deviation between subjects derived from the mean per-subject parameter value. Note that in 9 of the 60 datasets, the value of $T_{2a}^{*}$ (3 occurrences) or $T_{2e}^{*}$ (6 occurrences) converged on the upper fitting boundary of $100 ms$.

**References**

1. Gudbjartsson H, Patz S. The rician distribution of noisy mri data. Magn. Reson. Med. 1995;34:910–914. doi: 10.1002/mrm.1910340618.

2. Sati P, van Gelderen P, Silva AC, Reich DS, Merkle H, De Zwart JA, Duyn JH. Micro-compartment specific T2* relaxation in the brain. Neuroimage 2013;77:268–278. doi: 10.1016/j.neuroimage.2013.03.005.

3. Van Gelderen P, De Zwart JA, Lee J, Sati P, Reich DS, Duyn JH. Nonexponential T2* decay in white matter. Magn. Reson. Med. 2012;67:110–117. doi: 10.1002/mrm.22990.

4. Wharton S, Bowtell R. Fiber orientation-dependent white matter contrast in gradient echo MRI. Proc. Natl. Acad. Sci. 2012;109:18559–18564. doi: 10.1073/pnas.1211075109.
